# The Influence of Urban Park Attributes on User Preferences: Evaluation of Virtual Parks in an Online Stated-Choice Experiment

**DOI:** 10.3390/ijerph18010212

**Published:** 2020-12-30

**Authors:** Esther van Vliet, Gamze Dane, Minou Weijs-Perrée, Eveline van Leeuwen, Mayke van Dinter, Pauline van den Berg, Aloys Borgers, Kynthia Chamilothori

**Affiliations:** 1 Built Environment and Industrial Engineering & Innovation Sciences, Eindhoven University of Technology, 5612 AZ Eindhoven, The Netherlands; g.z.dane@tue.nl (G.D.); m.weijs.perree@tue.nl (M.W.-P.); m.h.w.v.dinter@tue.nl (M.v.D.); p.e.w.v.d.berg@tue.nl (P.v.d.B.); a.w.j.borgers@tue.nl (A.B.); k.chamilothori@tue.nl (K.C.); 2 Urban Economics, Wageningen University & Research, 6708 KN Wageningen, The Netherlands; eveline.vanleeuwen@wur.nl

**Keywords:** urban parks, virtual environment, stated-choice, park attributes, user preferences

## Abstract

Urban green areas, such as parks, are becoming increasingly important in densifying cities. Urban parks encourage physical and social activity, recreation and relaxation, and thus eventually promote people’s well-being. The aim of the current study is to examine which urban park attributes influence the preferences of park users, in order to offer recommendations regarding how urban parks of quality can be designed. To elicit the preferences of park visitors we designed an online stated-choice experiment. Seven park attributes, in particular the number and composition of trees and the presence of benches, side paths, a playground, litter, and flowers, were manipulated in a virtual park. In an online stated-choice task, videos of these park alternatives were presented and the preferences of 697 participants were measured. It is found that especially the number of trees and the presence of flowerbeds, particularly with a diversity of flowers, influenced participants’ preferences. The presence of many benches and a playground were valued as well, but to a lesser extent. The presence of litter was found to be less troublesome than expected. Alternatives with all trees placed in one cluster were disliked. Moreover, significant standard deviations were found for the presence of side paths, a playground, and the absence of litter, which indicates that preference heterogeneity for these attributes exist. In a latent class analysis, two groups were identified, namely a Nature-loving group, who mainly valued the trees and the flowers, and an Amenity-appreciating group, who valued almost all attributes. It can be concluded that natural elements and a variety of flower species are important in an urban park, while facilities are evaluated differently by different groups of people. These findings may support park designers and policymakers in decision-making. Moreover, it illustrates the usefulness of creating a virtual park in environmental preference research.

## 1. Introduction

The world population keeps expanding and this growth is mainly centralized in urban areas. It is expected that the percentage of the population living in urban areas will increase from 55 percent in 2018 to 68 percent in 2050 [1]. Urbanization poses challenges on the living environment of citizens and thus on their well-being. It can lead, among others, to crowding, crime, traffic problems, and poor housing conditions [2,3], which, in turn, can negatively influence people’s mental and physical health [4,5,6]. In addition, the World Health Organization has declared stress as the health epidemic of the 21st century [7]. Hence, living and working in an environment that allows for stress recovery is increasingly important.

Another problem that cities are facing is climate change and the resulting extreme weather conditions [8] and decreased biodiversity [9]. One way to deal with this is by greening the city, i.e., introducing and maintaining green spaces. Trees and plants mitigate climate change by reducing air pollution [10]. Green spaces can store and absorb rain during heavy rain falls and act as a cool spot during hot periods [11]. Moreover, biodiversity can be increased by creating diverse green areas [12]. Furthermore, urban green areas have, in addition to ecological and economic benefits, several social and health benefits [13]. The literature has shown that a green environment, i.e., an environment with greenery, positively influences the well-being and quality of life of citizens [14]. A green living environment reduces stress [15], encourages physical activity [16], and improves social cohesion [17]. The importance of green spaces became especially evident during the COVID-19 pandemic, when parks were one of the few public spaces one could visit during the lockdown measures. Recent research at an urban scale level found positive associations between restrictive measures for social gathering and park use [18], demonstrating the importance of urban parks for citizens during the COVID-19 pandemic.

In order to maintain and further promote park use, it is essential to create green spaces that are attractive to park users. In the last 25 years, a substantial body of work in environmental psychology has examined the dimensions contributing to landscape preference and identified favorable park attributes, such as spaced placement of trees on a smooth ground [19,20]. Nevertheless, there exist multiple examples of current parks, especially at the neighborhood level, which simply consist of a large open grass field with a few trees, that are not frequently visited by citizens [21]. Existing guidelines for the UK “Green Flag Award”, a distinction aimed at promote best practices in park design and management, also evaluate attributes such as the presence and maintenance of park facilities, biodiversity in the flora and fauna, or the presence of paths [19]. However, little is known about the importance of these different elements in creating high-quality urban green spaces.

The current study aims to address this knowledge gap by investigating the importance of different park elements in affecting the preferences of park users. The focus of the current study is on green urban parks, as they have an additional recreational and social purpose, compared to for example small urban greenery or green walls [22]. By analyzing user preferences towards parks, the importance of certain elements can be derived. These findings can be translated into guidelines for policy makers and park designers and managers.

To investigate how green urban parks could be improved, first a literature review is performed, which is presented in the section below. The third section presents the method and materials used in the present study, where virtual environments were used to examine park preferences with a high control over the presented park attributes. Next, the fourth section presents the data and results, followed by the discussion of the outcomes, limitations and recommendations in the fifth section. The final section presents the conclusions of the study.

## 2. Literature Review

Although the population in cities is growing [1], people have a preference for nature over urban areas [23]. People living in an area with urban green report fewer mental health problems [15], are more likely to exercise [15,19], more likely to socialize [17], and experience less noise annoyances [24]. Therefore, it is important that residents have access to parks in their neighborhood. The next sections present existing literature on the evaluation of parks, as well as on methods to examine environmental preferences.

### 2.1. Park Attributes and Preferences

There is substantial literature that has investigated park use and park preferences. In the literature there are various recurring attributes that play a role in explaining park preferences, which are size, distance, type and density of green, facilities, other people, maintenance, and noise. It is found that there is a trade-off between size and accessibility, with larger, more proximate parks being preferred [25]. Nevertheless, size and distance are less important as long as residents can access a park of at least 0.3 hectares [26] within one kilometer from their home [27,28]. On greenery, the literature showed that grass and trees are preferred over bushes and flowers [26,29] and that the density of the vegetation should be medium, i.e., not too dense and not too open [30,31,32]. This is in line with the landscape preference framework of Kaplan et al. [33], which explains that a landscape should be understandable for people, but it should also allow for exploration. Moreover, water elements are preferred [26,34], although it is expected that they are less important for satisfaction than the presence of grass and trees [29,35,36]. Nevertheless, water elements are an important aspect of climate robust urban design [37]. In addition, the presence of playgrounds, paths, and seating areas are valued by park visitors [38,39,40]. As parks have a social function, it is not surprising that the presence of other people in a park may influence people’s perception of a park. It is found that people value the presence of others, as long as they don’t perceive the park as being crowded [29,38]. It seems likely that the restorative effects of visiting a park can be enhanced by natural sounds. However, research mainly found evidence for the dislike of loud traffic and mechanical sounds, but no strong effects of natural sounds are found [41,42]. The last aspect that is found to influence preferences is the condition of the park [36,43]. Parks that are poorly maintained and have a lot of litter and dog excrement are disliked [38,41]. Although this hints at some ordering of the attributes based on importance, there is not a conclusive ranking of attributes in the literature.

Personal characteristics may also influence the preferences towards parks. It is found that age can play a role in preferences and park use, with people between 35 and 50 visiting a park most often [29,44]. Moreover, higher educated people have been shown to appreciate green spaces more [30,45]. Regarding gender, there are mixed findings. Some studies found no effect of gender on park preferences [30,45], while one study found that women had a more positive attitude towards green [46], and another study showed that men gave higher park preference scores [32].

While the literature shows a number of park attributes which are important for park preferences, little is known about the relative importance of these attributes. Some studies investigated this, but their results are not conclusive [26,29,38,41]. Moreover, they either used verbal descriptions [29,38] or static images [26,41], but none made use of virtual environments. This leaves a knowledge gap regarding the relative importance of park attributes, which can be measured using the upcoming methods of virtual environments, as explained next.

### 2.2. Investigating Environmental Preferences

Environmental preferences can be measured by using different research approaches, varying in tasks and representations of the environment. Most of the aforementioned studies on park preferences used a qualitative approach and performed interviews with park visitors on site. Some studies used a quantitative approach and let participants evaluate verbal descriptions [29,38] or visual images of (elements in) a park [41]. The advantage of visual stimuli over verbal descriptions is that it is less prone to variation due to individual interpretations [47]. A more advanced method is using virtual environments. It is found to give more reliable results than verbal descriptions [48,49] and still images [50].

A virtual environment can be shown in an immersive or non-immersive way. People feel more present in the environment when using immersive virtual reality (VR) [51], but this method is also more time-consuming. Non-immersive VR can be incorporated in an online survey. In this way a large and varied sample can be reached. Numerous studies used non-immersive virtual environments to measure landscape preferences (e.g., [48]). Research showed that psychological responses towards a non-immersive VR environment are comparable to psychological responses induced by a real environment [52]. Moreover, people evaluate non-immersive virtual environments as a realistic representation of the real world [53,54]. The most common method to present a virtual environment is by means of animated walk-through videos of approximately 30 s (e.g., [55]).

Different experimental tasks can be designed in which these virtual environments can be shown, such as a rating, ranking, or stated-choice task [56]. A stated-choice task is preferred, because it draws on the solid random utility theory [56,57]. Random utility theory explains that people’s choices can be predicted based on the overall utility that the alternatives offer plus a random component, i.e., people choose the alternative with the highest utility score. The theory assumes that people derive utility from each attribute and the overall utility of the alternative is the sum of the utility of the attributes [57].

### 2.3. Problem Statement

Urban green areas, such as parks, are important in a healthy living environment. Although the literature highlights elements that are important in an urban park, quantitative research on the relative importance of these attributes is scarce and inconsistent. Therefore, the present study seeks to answer the following research question: “Which park attributes are most preferred and which level of each attribute is favored?”. Since it is shown that personal characteristics might influence park preferences, the effect of this is also examined.

This question is answered by using an online stated-choice approach with virtual environments. The literature showed that virtual environments are a useful method to present an environment. A non-immersive form was selected, since in this way a large and varied sample size could be reached. Moreover, an online survey could be distributed during the COVID-19 pandemic, while executing an immersive VR experiment in a VR lab environment was not possible.

## 3. Materials and Methods

An online stated-choice task was designed in which preferences regarding park attributes were measured. The important park attributes, as found in the literature, were discussed with thirteen experts in the field of urban greenspace management and design. Experts included public space designers of the municipality of Eindhoven, students and researchers of the Eindhoven University of Technology and Wageningen University & Research, and employees of Planterra (Leusden, The Netherlands), a consultancy company on managing public space. Based on the feedback from the experts, seven relevant park attributes were selected to be manipulated in a virtual park. These are: number of trees, composition of trees, public furniture, cleanliness, paths, playground, and biodiversity. The attributes either had two or three levels: number of trees (few-some-many trees), composition of trees (trees spread-multiple tree clusters-one tree cluster), public furniture (some benches-many benches), cleanliness (no litter-some litter-much litter), paths (main path-main path and side paths), playground (absent-present), and biodiversity (no flowerbeds-single-species flowerbeds-multi-species flowerbeds). By varying the levels, according to an orthogonal experimental design [58], sixteen alternative park designs were created, shown in Table 1.

### 3.1. Design of Virtual Park

**Figure 1:**
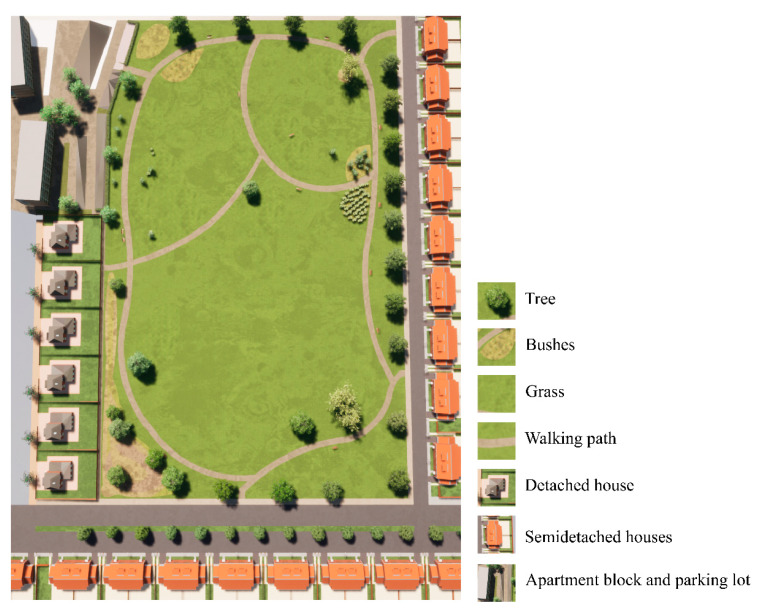
Top view of the baseline park.

A virtual park was created with the use of the software SketchUp 2018 [59] and Twinmotion 2020 [60]. The baseline design of the park was created in SketchUp, consisting of a neighborhood park of around 3.5 hectares surrounded by semi-detached and detached houses, and three apartment blocks. Surface materials, vegetation, and animated people were added in Twinmotion. The equipment of the playground and the litter were imported in Twinmotion from SketchUp models available in the 3D Warehouse repository [61]. Figure 1 shows a top view of the baseline park.

Apart from the baseline trees, the number of trees (beeches and birches) varied from 10 (‘few’) to 30 (‘some’) to 90 (‘many’). These trees could be either placed in one large cluster (‘one tree cluster’), in multiple smaller clusters (‘multiple tree clusters’), or spread across the park (‘spread’). Regarding public furniture, there are four benches visible in the videos of the park variations with ‘some benches’ and nine in the ‘many benches’ level. Regarding cleanliness, there could be no litter at all (the ‘no litter’ level), some litter or much litter (cans, candy wraps, beer and wine bottles) in and around the benches and spread across the park (the ‘some litter’ and ‘much litter’ level). There is one main path in all park variations and three additional side paths on either side of the main path for the ‘side paths’ alternatives. The playground consists of a climbing rack and a slide, a swing, and a seesaw. This is all absent in the alternatives with ‘no playground’ and present in the alternatives with a ‘playground’. Last, regarding flowers, there are alternatives with no flowerbeds at all (‘no flowers’), with three flowerbeds, but all with one type of flower (field scabious)(‘single-species flowerbeds’), and with again three flowerbeds, but with four different types of flowers (field scabious, ling heather, yarrows, and poppies)(‘multi-species flowerbeds’).

**Figure 2:**
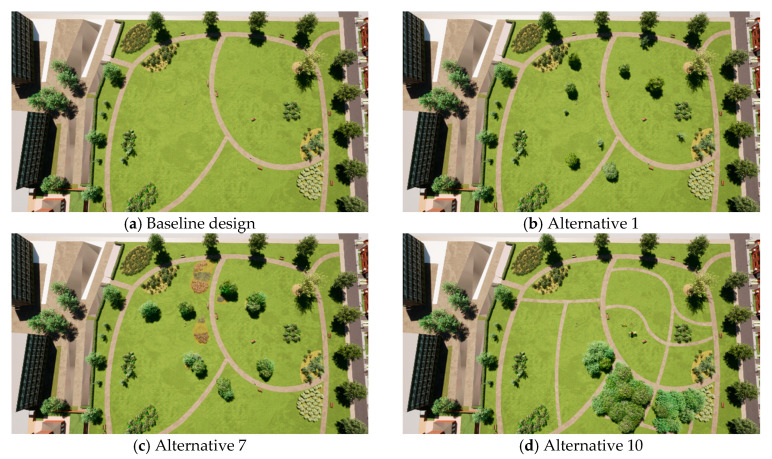
Top views of base level and alternatives; (**a**) Base level; (**b**) Alternative 1 showing few trees spread; (**c**) Alternative 7 showing some trees placed in multiple clusters and diverse flowerbeds; (**d**) Alternative 10 showing many trees placed in one cluster and side paths.

Figure 2 shows the top views of alternative park designs where the various amounts of trees and compositions can be seen, namely the baseline level, few trees spread across the park (alternative 1), some trees placed in multiple clusters (alternative 7), and many trees placed in one cluster (alternative 10). Moreover, one can see the flowerbeds with various types of flowers in alternative 7 (left of the path in the center) (Figure 2c) and the side paths in alternative 10 (Figure 2d).

**Figure 3:**
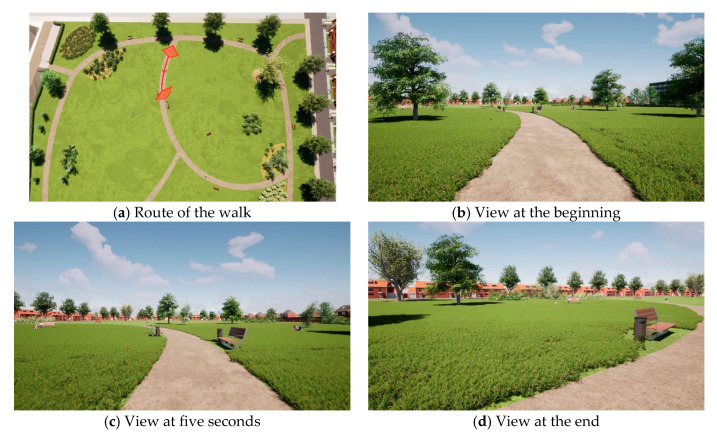
(**a**) Top view of the route of the walk; (**b**) Screenshot of video at the beginning; (**c**) Screenshot of video at five seconds; (**d**) Screenshot of the end of the video.

For each alternative, a video of 26 s in duration was created, which simulated a walk through the park. One of the sixteen videos is added as Supplementary Material. Figure 3 shows the route of the walk and a screenshot of the view at the beginning, at five seconds, and at the end of the video.

### 3.2. Design of Online Survey

The videos of the alternatives were presented in a stated-choice task, which was incorporated in an online survey made in the web server-based software LimeSurvey [62].

The sixteen videos were exported from Twinmotion as mp4 files in full HD (1920 × 1080 pixels), uploaded on YouTube and embedded in the LimeSurvey questionnaire. The embedded videos did not play automatically in the survey, so participants needed to press the play button for each video. The videos played with an automatically selected resolution depending on participants’ personal settings. Therefore, they were instructed to put the resolution of each video on the highest quality, before watching the video.

To avoid fatigue problems, each participant was presented with four choice tasks, corresponding to four pairs of alternatives randomly selected out of the sixteen alternatives.

Before the conduction of the survey, a pilot study was executed among a convenience sample of 10 respondents to test for ambiguity and the duration of filling out the questionnaire. Based on this, some questions were rephrased or redesigned in order to make them clearer.

### 3.3. Participants

The survey was approved by the Ethical Review Board of the Built Environment Department of Eindhoven University of Technology. The survey was conducted in July and the beginning of August 2020. It was distributed among the Digipanels, a group of residents that volunteers to participate in research, of two medium-sized Dutch cities (Eindhoven and ‘s-Hertogenbosch), and on social media. In the end, 739 participants gave their consent and completed the survey. The sample consisted of 376 participants living in Eindhoven, 228 participants from ‘s-Hertogenbosch, and 135 participants living elsewhere. The participants could enter a raffle, in which they had a chance of winning one of the ten gift cards worth 25 euros.

### 3.4. Procedure

An invitation to participate in the survey was distributed via mail and social media. At the beginning of the survey, participants were informed about the study objectives and their consent was asked.

The respondents were first giving an explanation before continuing with the four choice tasks. It was explained which elements could vary in the park scenes and it was made clear that the respondents should consider a neighborhood park that is close by. They were also instructed to put the quality of each video on the highest resolution.

The choice tasks were presented one by one. The two videos were presented next to each other. They were asked the following question each time: “Please watch both videos until the end. Which park would you prefer to visit?”. If they had no preference, they could select the ‘no preference’ option. There was no time limit.

The survey included also questions on park use, which concerned a related study. The questionnaire ended with questions regarding well-being and socio-demographics. In particular, participants were asked to rate their general health (based on the SF-36 [63]) and their well-being on five statements (based on the scale of [64]). The socio-demographic questions regarded gender, age, education, ethnic background, occupation, income, household composition, age of children if applicable, and possible disabilities.

### 3.5. Statistical Analysis

The data are analyzed with Stata/IC 16.1 [65] using a multinomial logit (MNL) model [57]. In order to take heterogeneity into account, also a mixed multinomial logit (ML) model [56] and a latent class (LC) model were run [66]. The ML model highlights parameters with a significant standard deviation, i.e., variables with a significant preference variation. The LC model is estimated to identify groups with similar preferences. With logistic regression, personal characteristics were linked to the identified groups. McFadden’s rho-square is estimated as an indicator of the goodness-of-fit of the estimated models [67]. The attribute levels are dummy coded to measure their effects. Since there are four attributes with three levels and three attributes with two levels, eleven parameters must be estimated. Besides, the parameter of the ‘no preference’ alternative, i.e., the constant, must be estimated. Thus, in total twelve parameters are estimated.

## 4. Results

Before conducting the statistical analysis, the data was cleaned. Participants that completed the survey within an implausible short period were removed. Respondents that only had to complete the choice task and the well-being and demographic questions and who finished the survey within four minutes were removed. For the participants that had to fill in the whole survey, so including questions about their park use, a threshold of nine minutes was set. Because of these time thresholds, 29 responses were dropped. Furthermore, respondents that selected only the ‘no preference’ option in the choice tasks were removed. This led to a drop of 13 responses.

The following sections describe the resulting sample, the results of the multinomial and the mixed multinomial logit models, and lastly the outcomes of the latent class model and the characteristics of the identified classes.

### 4.1. Sample

After cleaning the data, the sample consists of 697 respondents, of which 299 are female. The age ranges from 18 to 94 (MEAN = 51, SD = 18). Table 2 shows the socio-demographic characterization of the sample. Most of the respondents are Dutch (78%). One third of the sample works full-time (29%) and one third is already retired (30%). The rest works part-time, varying from working less than 12 h to working max. 35 h a week. The division of the net yearly income is roughly equal, with 30 percent earning less than €30.000 a year, 30 percent earning between €30.000 and €50.000, and 20 percent earning more than €50.000 a year. Another 20 percent preferred not to answer the question. Two fifths of the participants have a bachelor’s degree, while one fifth has a master or doctorate degree and one fifth has an MBO degree, which is equivalent to junior college education. The household of almost half of the sample consists of a couple without children. A quarter of the respondents is either somewhat or extremely limited by a disability.

Besides questions on personal characteristics, the participants were asked to rate their health and well-being. Health was measured on a five-point scale [63]. The average health score is 3.46 (SD = 0.91). Well-being was measured with five statements (α = 0.91) on a seven-point scale [64]. The average score is 5.09 (SD = 1.23).

### 4.2. Mixed Multinomial Logit Model

The MNL model and the ML model were estimated with Stata/IC 16.1 [65]. The estimated parameters of both models showed similar relationships, with the ML model having a slightly higher goodness-of-fit (pseudo $\rho^{2}$ = 0.30, versus MNL pseudo $\rho^{2}$ = 0.28). Moreover, the ML showed that there are some parameters that had a significant standard deviation. As the MNL parameters are similar to the mean parameter estimates of the ML model, we will only discuss the results of the ML model.

**Figure 4:**
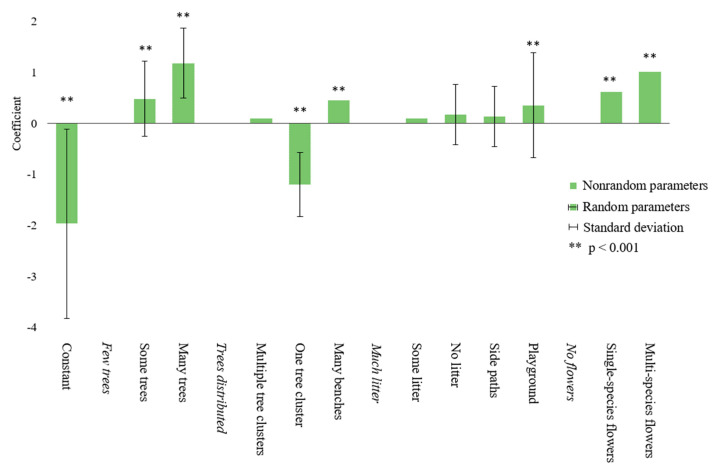
Diagram of the influence of the elements on preferences of the ML model. Elements with a significant random parameter are indicated by error bars, representing one standard deviation above and under the mean.

The ML model is estimated with the assumption that the parameter estimates follow a normal distribution. Table 3 shows the results of the ML model in detail. The standard deviation is shown for the parameters for which it was significant. Figure 4 shows the coefficients related to each element in a diagram. The standard deviations of the elements with a random parameter are included as error bars, ranging from one standard deviation below the mean to one standard deviation above the mean.

The model shows that only the constant, i.e., the ‘no preference’ option, and the alternatives with trees positioned in one cluster have a negative parameter (respectively, β = −1.97, *p* < 0.001, and β = −1.2, *p* < 0.001). This means that participants were unlikely to choose the ‘no preference’ option, nor were they likely to choose the alternatives with the trees placed in one cluster. Indeed, of the 697 respondents only 146 respondents selected at least once the ‘no preference’ option.

The element ‘many trees’ has the most positive influence on the preferences (β = 1.18, *p* < 0.001), followed by the element ‘multi-species flowerbeds’ (β = 1.01, *p* < 0.001). Next, the alternatives with ‘single-species flowerbeds’ (β = 0.62, *p* < 0.001) and with ‘some trees’ (β = 0.48, *p* < 0.001) were often chosen. The influences of ‘many benches’ and of the ‘playground’ are comparable and somewhat lower than the influence of the natural elements (respectively β = 0.45, *p* < 0.001 and β = 0.35, *p* < 0.001). The elements ‘trees positioned in multiple clusters’ (β = 0.10, *p* = 0.42), ‘no litter’ (β = 0.17, *p* = 0.12), ‘some litter’ (β = 0.09, *p* = 0.41), and ‘side paths’ (β = 0.13, *p* = 0.09) do not significantly influence preferences.

The results show that the standard deviations of the constant (SD = 1.84, *p* < 0.001), and of the elements ‘some trees’ (SD = 0.78, *p* < 0.001), ’many trees’ (SD = 0.76, *p* < 0.01), ’trees placed in one cluster’ (SD = −0.65, *p* < 0.01), ’no litter’ (SD = 0.64, *p* < 0.01), ’side paths’ (SD = 0.61, *p* < 0.1), and ’playground ’ (SD = 1.08, *p* < 0.001) were significant. Thus, for these elements it may be assumed that the parameters vary from one individual to another.

### 4.3. Latent Class Model

**Figure 5:**
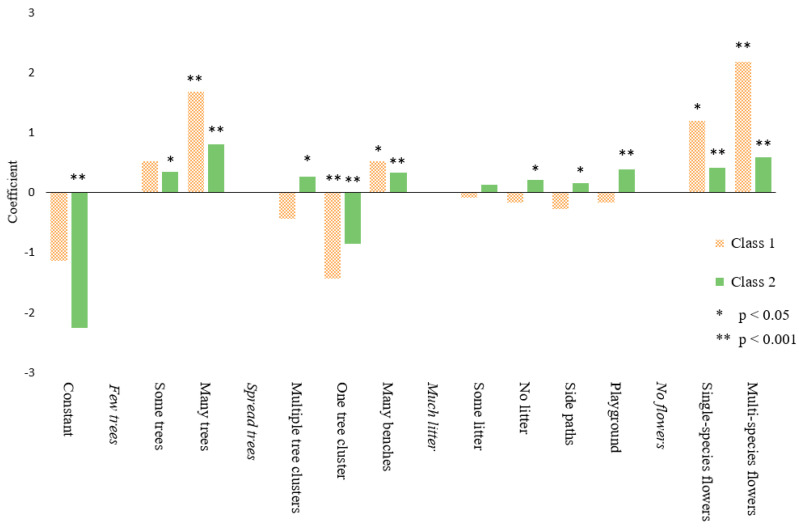
Diagram of the results of the LC model, which shows the influence of the elements on preferences for class 1 and class 2. Class 1 is indicated by dotted orange bars, while class 2 is indicated by solid green bars.

Latent class models were estimated with two, three, and four classes. Based on the Bayesian Information Criterion [68], the model with two classes was selected (pseudo $\rho^{2}$ = 0.31). There were 108 respondents in class 1 versus 589 respondents who belonged to class 2. The parameters that explained the preferences for each class, differed on various aspects. In Table 4, the estimated values by the LC model are shown, while Figure 5 shows the results of the LC model graphically.

For the first class, the elements ’many trees’ (β = 1.68, *p* < 0.001), ’many benches’ (β = 0.52, *p* < 0.05), and both ’single-species flowers’ and ’multi-species flowers’ (respectively β = 1.2, *p* < 0.01, and β = 2.18, *p* < 0.001) have a positive influence on the preferences, while trees positioned in one cluster (β = −1.43, *p* < 0.001) negatively influences the preferences. For the second class, all elements have a significant effect on the preference, except for ’some litter’ (β = 0.13, *p* = 0.20). Of these significant elements, in line with the MNL and ML model, only the constant and the trees placed in one cluster have a negative effect (respectively β = −2.26, *p* < 0.001, and β = −0.86, *p* < 0.001).

The two classes show that there is one group of respondents that seem to be less pronounced in their preferences. For this class, class 2, almost all elements have some effect. Therefore, henceforth class 2 is labelled the amenity-appreciating class. The other group of respondents, class 1, specifically values many trees and diverse flowerbeds; this class will be referred to as nature-loving class. It is interesting to see whether personal characteristics of the groups can be identified. In the next section the influence of these characteristics is discussed.

### 4.4. Class Membership and Personal Characteristics

The probability for each respondent to belong to the nature-loving class and the probability to belong to the amenity-appreciating class were generated by the LC model. Respondents were assigned to the class with the highest probability. Participants were on average more likely to belong to class two (Pr = 0.82, SD = 0.30) than class one (Pr = 0.18, SD = 0.30).

A logistic regression was run with the class assignment as dependent variable and the personal characteristics (age, gender, disability, well-being, health, household composition, education, occupation and income) as independent variables. Non-significant parameters were left out resulting in the model shown in Table 5. Correlations between the characteristics were inspected, but no problematic correlations were identified.

Respondents that are older are more likely to belong to the nature-loving class (β = 0.03, *p* < 0.001). Moreover, respondents with a disability (β = 0.18, *p* < 0.05), with a Dutch nationality (β = 1.3, *p* < 0.001), with a high education (β = 0.17, *p* < 0.05), and with a high income or who did not prefer to say what they earned (respectively β = 0.17, *p* < 0.001 and β = 0.57, *p* < 0.001) are more likely to belong to the nature-loving class. Female respondents (β = −0.51, *p* < 0.001), respondents with a higher health score (β = −0.13, *p* < 0.001), with a household with children (β = −0.72, *p* < 0.001), and who have a part-time job (β = −0.23, *p* < 0.05) are more likely to belong to the amenity-appreciating class. Last, the negative constant (β = −3.4, *p* < 0.001) confirms the high membership probability related to the amenity-appreciating class.

## 5. Discussion

The MNL and ML model highlight similar relationships. In the next sections, the average influence of the elements on preferences is discussed first, followed by a closer look at the parameters with a random distribution. The classes as found by the LC model are discussed, as well as the personal characteristics that can be linked to the classes. The final section presents an overview of the limitations and recommendations for further research.

### 5.1. Average Influence of Elements on Preferences

Both the MNL and ML model show that the largest parameter is related to the constant and negative. It shows that participants were very unlikely to choose the ‘no preference’ option. They noticed differences between the alternatives and had a preference of one alternative over the other.

The element ’many trees’ had the largest positive influence on preferences. The element ’some trees’ also influences preferences positively, but to a lesser extent. This is in line with the studies of Arnberger and Eder [41] and Nordh et al. [29] and expresses the appreciation of natural elements. People prefer a semi-open space over a dense landscape [30,31,38]. The alternatives with ’many trees’, despite the increase in trees, still had a semi-open character. Another explanation has to do with the shadows. In the alternatives with ’many trees’, there were more shadows. The survey was conducted in the summer and it could be that people value these cool spots more. More hot periods are expected, due to climate change, and trees play an important role in creating cool places [69]. It can be concluded that more trees are preferred. Parks should thus be designed with a considerable number of trees while ensuring a semi-open character where there is space for recreation. Our manipulation with birches and beeches in a neighborhood park showed that around 60 trees per hectare is most preferred.

The second largest parameter is related to ’multi-species flowerbeds’. It is comparable to the parameter of ’many trees’, although somewhat lower. The single-species flowerbeds have the third largest influence, although the difference between this parameter and the parameter of ’multi-species flowerbeds’ and ‘many trees’ is slightly larger. The positive influence of flowers is in line with the research of Shr et al. [70], who showed that a high diversity was preferred, but contradicting to the study of Nordh et al. [29], who showed that flowers were one of the least important park elements. However, the latter study used verbal descriptions in the choice task. In the current study, the flowers add diversity in shapes and color to the scenes, especially for the diverse flowerbeds. Moreover, although not included in the current study, flowers can increase the biodiversity by attracting insects, which in turn can increase the presence of nature in an urban park.

Next, the elements ’many benches’ and ’playground’ influence the preferences positively and to a comparable extent. This confirms what is shown in literature, namely that facilities such as benches [39,40] and playgrounds [38] are valued. The current experiment showed that many benches (6 per hectare) are preferred over some benches (3 benches).

The results show that, although the Amenity-appreciating class prefers alternatives with no litter over alternatives with some or much litter, on average litter did not significantly influence preferences. This is contradicting to the negative attitude towards a lack of maintenance that is commonly expressed by people [36,43]. It also contradicts with a study of Arnberger and Eder [41] which showed that a high presence of litter and dog excrement was disliked, while a bit of little was preferred over no litter at all, with the same being true for dog excrement. It might be that people focused on the natural elements and did not notice the litter. Nevertheless, it indicates that the role of litter in park preferences is more limited than expected, especially when the amount of litter is not extraordinary.

The presence of side paths did not influence preferences. Research on trails did show that the width of the path significantly influenced park preferences [41]. In the current study, side paths were presented that connected to the main path. The camera movement continued along the main path however, in order to keep the videos comparable between different alternatives. Therefore, participants might not have experienced the sidewalks as possible walking routes. Moreover, the importance of side paths might depend on the reason of the visit, which was not made explicit in the current study.

The last attribute is the distribution of the trees. The influence of ’multiple tree clusters’ on preferences did not significantly differ from the influence of the trees being spread out, but alternatives with ’one tree cluster’ were significantly less liked than the alternatives with either trees spread or placed in multiple tree clusters. It would be expected that multiple tree clusters would be favored over the other compositions, because this creates an optimal mix of open and closed areas, which is, as mentioned before, preferred [30,31]. Apparently, the difference between alternatives with the trees spread or placed in multiple clusters was small and both retained a semi-open landscape. However, in the alternatives with trees placed in one cluster, there was a large open field, next to the tree cluster. This might explain why these alternatives were disliked. Moreover, the tree cluster was placed at some distance. Since people value the presence of trees [29], it makes sense that people prefer the trees spread or placed in multiple clusters, because in those alternatives the trees were closer by.

To conclude, especially the presence of trees and (diverse) flowerbeds is appreciated. These trees should be either spread across the park or placed in multiple clusters, at least not in one large cluster. Next, the presence of many benches and a playground are valued, but to a lesser extent. Preference heterogeneity might explain why these attributes are, on average, less valued. This heterogeneity is discussed next.

### 5.2. Preference Heterogeneity

The elements were tested with random parameters in the ML model and significant standard deviations, indicating preference heterogeneity, are found for the constant and the elements ‘some trees’, ‘many trees’, ‘one cluster’, ‘no litter’, ‘side paths’, and ‘playgrounds’.

The standard deviation of the constant runs from a just negative parameter to an extreme negative parameter (taking into account one unit of SD). The same holds for the element ‘one cluster’, with a standard deviation that runs from a large negative parameter to a considerable negative parameter. It indicates that in general, participants were not likely to select the ‘no preference’ option and alternatives with one tree cluster, but some people were considerably more hesitant to choose these than others.

For the element ‘many trees’, the influence varies from a somewhat positive effect to a large positive effect. Also, for the element ‘some trees’, the tastes are mostly positive. This indicates that most people prefer more trees, but for some people it is more important than for others.

Regarding the presence of litter, more than half of the participants prefer the absence of litter, while others dislike to some extent the absence of litter. For some people, litter had no influence at all. It might be that some participants did notice the litter and were bothered by it, while for others the litter might be an indicator of human presence, as was the case in previous research [41]. Further, some people might have focused on other aspects of the park and have taken no notice of the litter.

Regarding side paths and playgrounds, preference variations are not surprising either. People that visit a park to walk or people that visit a park with their children are likely to value these elements differently. For both elements, the standard deviation indicates a range between positive and negative preferences. This means that some people were likely to select alternatives where these attributes were present, while others were likely to select alternatives where these attributes were absent.

### 5.3. Identified Classes

Two classes were identified in the data, a nature-loving class and an amenity-appreciating class. The nature-loving class specifically values many trees and diverse flowers, while they dislike the trees positioned in one cluster. The amenity-appreciating class also values the natural elements and dislikes the one cluster of trees, but to a lesser extent. This explains the standard deviation of the elements ‘some trees’ and ‘many trees’, and ‘one cluster’, which was significant but in the case of ‘some trees’ and ‘many trees’ ranged from a somewhat positive to an extremely positive effect and for the ‘one cluster of trees’ from a somewhat negative to a very negative effect. Surprisingly, for ‘single-species flowerbeds’ and ‘multi-species flowerbeds’ no significant preference heterogeneity was found, while the nature-loving class values these both considerably more than the amenity-appreciating class.

In addition to these natural elements, the amenity-appreciating class values the presence of furniture, side paths, and a playground and the absence of litter. It might be that this group visits the park for more different activities, while the nature-loving class mainly visits parks to simply be in nature. Moreover, trees placed in multiple clusters are significantly more valued than trees spread by the amenity-appreciating class and alternatives with one cluster of trees are less disliked by this class than by the nature-loving class. As explained earlier, a composition of multiple clusters or one tree cluster creates open spaces for recreation, which is in line with the hypothesis that the amenity-appreciating class might be valuing the possibility to perform different activities in the park.

### 5.4. Class Membership and Personal Characteristics

Although respondents are more likely to belong to the amenity-appreciating class, the probability of belonging to the nature-loving or amenity-appreciating class could be predicted by several personal characteristics.

Age plays a role, with older people being more likely to belong the nature-loving class. This class mainly values the flowers, which is in line with the findings of Nordh et al. [29], who found that people above 60 especially valued flowers. Moreover, the one facility that the nature-loving class does value is the presence of many benches. It is not surprising that people with a somewhat limiting or extremely limiting disability are more likely to belong to this class as well.

The current study also found an effect of gender, while other studies failed to find an effect [30,45]. An explanation for the finding that women are more likely to belong to the amenity-appreciating class could be that women might go more often to a park with their children and therefore value the presence of a playground. In fact, people with children are also more likely to belong to the amenity-appreciating class. Moreover, this class dislikes the presence of litter. Litter is found to be associated with crime [71] and it is found that women associate parks with crime [32]. This might explain why women are more likely to belong to the class that dislikes the presence of litter.

Regarding ethnicity, a significant effect was found with findings showing that Dutch people are more likely to belong to the nature-loving class rather than the amenity-appreciating class. This seems in line with the study of Buijs et al. [72], who showed that Dutch people favored wild nature images, while immigrants favored functional nature. However, the Dutch group in the current study includes native Dutch people, as well as immigrants with a double nationality. The non-Dutch group is too small and diverse to be able to conclude on effects of nationality.

The results of the current study support the common finding that more educated people appreciate green spaces more strongly [30,45]. People with a high education level are more likely to belong to the nature-loving class than people with a low or medium education level.

Next, the outcomes show that people with a part-time job are more likely to belong to the amenity-appreciating class than people with a full-time job or people who were unemployed or retired. An explanation for this could be that many students have a part-time job and they often visit a park for various reasons, from sporting to hanging out with friends. Moreover, people with children, especially women, often work part-time as well. Therefore, people with a part-time job are likely to belong to the class who values the facilities and the alternatives with open spaces. Indeed, younger people, females, and people with children are more likely to belong to this class as well.

Last, people with a high income or who preferred not to say how much they earned were found to be more likely to belong to the nature-loving class. An explanation for this is that people with a lower income probably have lower or no access to a private outdoor space and therefore visit a park for a variety of reasons [73]. They are likely to appreciate a playground, side paths and open spaces for recreation and thus it makes sense that they belong to the amenity-appreciating class.

### 5.5. Limitations and Recommendations

Although this works brings new insights on the influence of park attributes on preference, the limitations of the current study must be noted. Research showed that people do not evaluate real environments exactly the same as virtual environments [74], although studies did show that participants reviewed a virtual environment as a realistic representation of the real world [53,54]. One should be aware of the differences when translating the findings to real parks. Further research should examine if and how the preferences regarding a virtual park and a real park differ.

The abovementioned limitations should be taken into account when reaching any conclusions about the influence of maintenance on preferences. A virtual park on itself looks cleaner than a real park. Therefore, the presence or absence of litter might have a limited effect on this overall manicured image of the park. Nevertheless, the preferences of the respondents in the amenity-appreciating class were significantly influenced by the absence of litter, so at least for a part of the respondents the litter did disturb the clean image of the park. In further research, next to litter, dog excrement, graffiti or vandalized furniture could be added. Next, the influence of the maintenance of the greenery could be investigated. This can be done in a virtual park by manipulating the length of the grass, the presence of weeds, and the wilderness of the flowers. It also makes the virtual parks more realistic, easing the abovementioned limitation. Furthermore, it would be interesting to see how implementations of nature-based solutions for climate change, such as swales, influence the preferences, as this would provide insights on how nature-based solutions are evaluated by park users. Another topic of further research is the investigation of whether preferences differ when one visits a park during the day or in the evening, as research showed that some attributes of a virtual street were evaluated differently during day or nighttime [54]. Daylight conditions can be varied in a virtual park and preferences regarding different streetlight conditions can be investigated.

Besides comparing the results to preferences regarding a real environment, it is interesting to see whether preferences found in this non-immersive VR experiment are comparable to preferences in an immersive VR experiment. When using the same set up, the validity of both methods can be investigated. Literature is unclear on whether immersive or non-immersive VR is a more valid method to measure park preferences, although an immersive experiment is expected to increase the feeling of being present in the environment [51]. In immersive VR, the participants have more freedom to look and walk around and choose on what to focus. Therefore, it is advised to collect information on how the participant experienced the environment, what they noticed and what they paid attention to in the immersive VR experiment.

The current study only manipulated visual stimuli by varying the park designs presented to the participants. However, especially when employing an immersive VR experiment, a fourth dimension can be added as well in the form of odors or sounds, as research has shown that certain smells [36] and sounds [42] might influence park user preferences. In this way the influence of smell and noise on park user preferences could be investigated as well.

Although stated-choice experiments rely on a solid theory, the question remains whether participants encounter similar choice options in real life and if they would have the same preferences when there is no other park alternative. However, only 146 of the 697 respondents indicated that they had no preference for one park or the other. It shows that most participants did notice differences between park designs and had a preference for one alternative over the other. In the study of Bullock [38], who used verbal descriptions, the percentage of participants who did not have a preference for one park alternative over the other was way higher. This implies that realistic variations in an environment can be manipulated and measured with a virtual environment. In addition, it might indicate a high level of involvement possibly due to engaging videos.

Another limitation of using a stated-choice experiment is that there is a limit to the number of attributes that can be manipulated without inducing participant fatigue. Moreover, in this study, a single park design was used as a baseline, which limits the applicability of findings in other contexts. The outcomes of this study are thus a first step for systematic research in this direction, as multiple park configurations should be examined to test the generalizability of these findings across variations in landscape, neighborhood, or urban integration. While the current study allows systematic investigation of the effects of specific attributes on park preference and its findings can provide useful insights for park design and management, additional considerations regarding the urban, social, and geographical context (i.e., neighborhood characteristics, topography, network relations) are an essential part in the landscape design process. Moreover, the current study focused on neighborhood parks, but further research could investigate how preferences differ regarding parks of different sizes and at different locations. Based on that, different suggestions can be given to park designers, depending on the size and location of the to-be-designed park.

There were some limitations regarding the sample. First of all, the sample consisted of mainly Dutch people. In addition, most of the respondents had no children, or at least no children living at home. Moreover, in the current research, the purpose of the park visit was not specified, while it could be an influencing factor in how people evaluate parks. For further research it would be beneficial to determine the purpose of the visit for the participants or to ask participants to describe the purpose of their usual park visit.

Last, it must be noted that the survey was distributed during the COVID-19 pandemic. During the intelligent lock-down in the Netherlands, parks were one of the few places that one could visit. Hence, it is possible that people have a different association with parks than before the COVID-19 pandemic. Possibly, people are more aware of the importance of green spaces. This might explain why only few people, selected the ‘no preference’ option, which could indicate a high level of involvement. In addition, the survey was distributed during the summer. It might be that people evaluate a park differently during different seasons. It could be that people have a higher appreciation for trees during the summer, because of the shadows they provide. It would thus be interesting to perform the same experiment in a few years, when the situation around COVID-19 is stable, and during different seasons.

## 6. Conclusions

The present study investigated the relative importance of different park attributes (number of trees, composition of trees, public furniture, cleanliness, side paths, a playground, and flowers) in an online stated-choice experiment using animated videos of a walk through a simulated park. A total of 697 participants evaluated their preferred park alternative between four pairs randomly selected from a total of sixteen park alternatives with varying attributes. The results confirm that natural elements are valued by park users. In particular, it showed that the presence of many trees and multi-species flowerbeds have the largest positive influence on preferences, whereas parks with trees placed in one cluster are disliked. Moreover, the current study showed that some park elements, such as the presence of a playground and side paths and the absence of litter, are evaluated differently by different people. The respondents could be divided into a more nature-loving class and an amenity-appreciating class. Older people, males, people with a disability, with a Dutch ethnicity, with a higher education level, and with a high income or who did not want to say how much they earned, have an increased likelihood to belong to the nature-loving class. A variety of facilities, depending on the neighborhood characteristics, such as benches, a playground, open areas, and walking paths are important to create an appreciated park by the residents. The current study examined in a quantitative way what role the investigated park attributes play in explaining user preferences, in contrary to most studies on park preferences. Guidelines can be derived from these findings that can help policymakers and park designers in the decision-making process regarding park designs.

## Figures and Tables

**Table 1:** Sixteen alternative park designs and their corresponding attribute levels.

Alternative	Number of Trees	Composition of Trees	Public Furniture	Cleanliness	Paths	Playgrounds	Biodiversity
1	Few trees	Spread	Some benches	No litter	Main	None	None
2	Few trees	One Cluster	Many benches	Some litter	Side paths	None	Multiple
3	Few trees	Multiple clusters	Some benches	Much litter	Side paths	One	Single
4	Few trees	One Cluster	Many benches	Some litter	Main	One	Single
5	Some trees	Spread	Some benches	Some litter	Side paths	One	Single
6	Some trees	One Cluster	Many benches	No litter	Main	One	Single
7	Some trees	Multiple clusters	Some benches	Some litter	Main	None	Multiple
8	Some trees	One Cluster	Many benches	Much litter	Side paths	None	None
9	Many trees	Spread	Many benches	Much litter	Main	One	Multiple
10	Many trees	One Cluster	Some benches	Some litter	Side paths	One	None
11	Many trees	Multiple clusters	Many benches	No litter	Side paths	None	Single
12	Many trees	One Cluster	Some benches	Some litter	Main	None	Single
13	Some trees	Spread	Many benches	Some litter	Side paths	None	Single
14	Some trees	One Cluster	Some benches	Much litter	Main	None	Single
15	Some trees	Multiple clusters	Many benches	Some litter	Main	One	None
16	Some trees	One Cluster	Some benches	No litter	Side paths	One	Multiple

**Table 2:** Socio-demographic characterization of the sample.

Personal Characteristic	Category	Number of Respondents	Percentage
Gender	Female	299	43
	Male	398	57
Ethnicity	Dutch	546	78
	Non-Dutch	151	22
Occupation	Full-time	199	29
	Part-time	240	34
	Unemployed/retired	258	37
Net yearly income	Less than €30.000	200	29
	€30.000–50.000	216	31
	More than €50.000	147	21
	Prefer not to answer	134	19
Education	Low education	276	39
	High education	421	61
Household	With children	142	20
	Without children	555	80
Disability	Not disabled	526	75
	Disabled	171	25
	Total	697	100

**Table 3:** Results of mixed logit model. The predictors in italics indicate the base level of each attribute.

Predictor	β	*p*	SD	*p*
Constant	−1.97	<0.001	1.86	<0.001
*Few trees*				
Some trees	0.48	<0.001	0.74	<0.001
Many trees	1.18	<0.001	0.69	<0.05
*Spread*				
One tree cluster	−1.2	<0.001	0.63	<0.01
Multiple tree clusters	0.10	0.42		
*Some benches*				
Many benches	0.45	<0.001		
*Much litter*				
Some litter	0.09	0.41		
No litter	0.17	0.12	0.60	<0.01
*Only main path*				
Side paths	0.13	0.09	0.59	<0.01
*No playground*				
Playground	0.35	<0.001	1.03	<0.001
*No flowers*				
Single-species flowers	0.62	<0.001		
Multi-species flowers	1.01	<0.001		

**Table 4:** Results of latent class model. The predictors in italics indicate the base level of each attribute.

Predictor	Class One β	*p*	Class Two β	*p*
Constant	−1.14	0.06	−2.26	<0.001
*Few trees*				
Some trees	0.52	0.13	0.34	<0.01
Many trees	1.68	<0.001	0.81	<0.001
*Spread*				
One tree cluster	−1.43	<0.001	−0.86	<0.001
Multiple tree clusters	−0.44	0.26	0.27	<0.05
*Some benches*				
Many benches	0.52	<0.05	0.33	<0.001
*Much litter*				
Some litter	−0.087	0.80	0.13	0.20
No litter	−0.16	0.67	0.21	<0.05
*Only main path*				
Side paths	−0.28	0.27	0.16	<0.05
*No playground*				
Playground	−0.16	0.59	0.38	<0.001
*No flowers*				
Single-species flowers	1.2	<0.01	0.41	<0.001
Multi-species flowers	2.18	<0.001	0.59	<0.001

**Table 5:** Logistic regression predicting probability of belonging to class 1 (Nature-loving class) based on personal characteristics.

Predictor	β	*p*
Age	0.03	<0.001
Female	−0.51	<0.001
Disability	0.18	<0.05
Health	−0.13	<0.01
Dutch	1.29	<0.001
Household with children	−0.72	<0.001
High education	0.17	<0.05
Part-time job	−0.23	<0.05
Income kept private	0.57	<0.001
High income	0.33	<0.001
Constant	−3.37	<0.001

## Data Availability

Data available on request due to restrictions eg privacy or ethical. The data presented in this study are available on request from the corresponding author. The data are not publicly available due to privacy reasons.

## Supplementary Materials

The following are available online at https://www.mdpi.com/1660-4601/18/1/212/s1, Video S1: Video of alternative 7.
